# Modeling Species Distributions from Heterogeneous Data for the Biogeographic Regionalization of the European Bryophyte Flora

**DOI:** 10.1371/journal.pone.0055648

**Published:** 2013-02-11

**Authors:** Rubén G. Mateo, Alain Vanderpoorten, Jesús Muñoz, Benjamin Laenen, Aurélie Désamoré

**Affiliations:** 1 Institute of Botany, University of Liège, Liège, Belgium; 2 Real Jardín Botánico (CSIC), Madrid, Spain; 3 Azorean Biodiversity Group (CITA-A) and Platform for Enhancing Ecological Research and Sustainability (PEERS), Universidade dos Açores, Department Ciências Agrárias, Terceira, Açores, Portugal; 4 Universidad Tecnológica Indoamérica, Ambato, Ecuador; DOE Pacific Northwest National Laboratory, United States of America

## Abstract

The definition of biogeographic regions provides a fundamental framework for a range of basic and applied questions in biogeography, evolutionary biology, systematics and conservation. Previous research suggested that environmental forcing results in highly congruent regionalization patterns across taxa, but that the size and number of regions depends on the dispersal ability of the taxa considered. We produced a biogeographic regionalization of European bryophytes and hypothesized that (1) regions defined for bryophytes would differ from those defined for other taxa due to the highly specific eco-physiology of the group and (2) their high dispersal ability would result in the resolution of few, large regions. Species distributions were recorded using 10,000 km^2^ MGRS pixels. Because of the lack of data across large portions of the area, species distribution models employing macroclimatic variables as predictors were used to determine the potential composition of empty pixels. K-means clustering analyses of the pixels based on their potential species composition were employed to define biogeographic regions. The optimal number of regions was determined by v-fold cross-validation and Moran’s I statistic. The spatial congruence of the regions identified from their potential bryophyte assemblages with large-scale vegetation patterns is at odds with our primary hypothesis. This reinforces the notion that post-glacial migration patterns might have been much more similar in bryophytes and vascular plants than previously thought. The substantially lower optimal number of clusters and the absence of nested patterns within the main biogeographic regions, as compared to identical analyses in vascular plants, support our second hypothesis. The modelling approach implemented here is, however, based on many assumptions that are discussed but can only be tested when additional data on species distributions become available, highlighting the substantial importance of developing integrated mapping projects for all taxa in key biogeographically areas of Europe, and the Mediterranean peninsulas in particular.

## Introduction

The definition of biogeographic regions at different spatial scales based upon their biotic composition has most recently gained increasing attention to provide, through the statistical analysis of distribution data, a spatially explicit framework for a range of basic and applied questions in historical and ecological biogeography, evolutionary biology, systematics and conservation [1]. Given that species evolve within areas from which they may subsequently disperse, the taxonomic composition of an area’s flora and fauna reflects the degree to which it acts as a centre of origin, has been colonized by dispersing organisms, or has been subject to large-scale forces [2]. This raises the question of whether, as opposed to the early view of common plant kingdoms and animal regions, biogeographic patterns are taxon-specific. In particular, Cox [3], [4] emphasized the relevance of dispersal ability to biogeographical patterns, pointing out that mammals are unusual, even within the animal kingdom, for their very limited ability to disperse across an ocean barrier. He therefore suggested two basic patterns of global biogeography, one for groups with a high dispersal ability (such as flowering plants), and one for those with low dispersal ability (such as mammals). Recent meta-analyses of multiple groups of European seed plants and animals resolved, however, spatially coherent clusters [5]. Similar geographic regions were resolved from the analyses of plant and mammal taxa, suggesting that the distributions of these groups are controlled by the same environmental variables and that environmental forcing is sufficient to erode the signature of taxon-specific differences in life-history traits [6]. The main differences observed among groups were the number of regions identified, which was associated to species mean range size and dispersal ability [5].

The primary aim of the present study was to produce a biogeographic regionalization of Europe from bryophyte species distributions. Bryophytes are a group of early land plants that exhibit specific eco-physiological features and life-history traits coupled with unique diversity and distribution patterns [7]. For instance, bryophyte diversity patterns do not fit with a latitudinal gradient of decreasing species diversity from the tropics towards the poles [8], [9], [10], a pattern traditionally considered one of the few truly general ones in biogeography and ecology [11]. While many of the floristic elements and distribution centres of southern African bryophytes correspond to those described for seed plants [12], the distribution patterns of bryophytes, fern, and seed plant species in the UK were shown to markedly differ and reflect their contrasting rates of evolution, centres of diversity, dispersal ability and ecophysiology [13]. Because of their poikilohydric condition, bryophytes thrive in moist environments that are not necessarily the richest for other organisms with different adaptive strategies to drought. Bryophyte also typically exhibit, owing to their high dispersal capacities, spatially disjunct distributions [10]. This suggests that the analysis of bryophyte species distribution at the European scale could result in large, not necessarily spatially coherent groups, in agreement with the observation that, at the molecular level, mosses best fit the pattern ‘everything is everywhere’ [8].

Knowledge of the distribution of European bryophytes is uneven, however, ranging from complete atlases to more sporadic information found in checklists, local floras, databases and herbaria. This issue, which is common with distribution data, has been variously dealt with [14], [15]. Linder et al. [16] conservatively extrapolated range maps from point data for all except the rarest species. Here, we employed species distribution models (SDMs), which have become a powerful tool to generate maps of potential distribution, or ecological suitability, in areas where distribution information is scarce or lacking [17]. A major assumption of SDMs is, however, that data for each species represent a random sample of their distribution in the area of study so that the frequency of a species in an area reflects its actual macroclimatic preference and not a sampling bias [17], [18], [19]. We circumvented the potential issue of model bias associated with the spatial heterogeneity in the distribution of available data by subsampling the data from the most intensively surveyed areas, as advocated by Araújo & Guisan [14] and Hijmans & Elith [20]. We then stacked potential distributions to identify biogeographic regions and indicator species for each region, assessing the impact of different data subsampling schemes. We finally tested the hypothesis that, owing to the high dispersability and specific eco-physiology of bryophytes, the analysis of their distributions resolves fewer regions that do not coincide with those observed in vascular plants.

## Materials and Methods

### Species Data

Bryophyte species distributions were recorded using Military Grid Reference System (MGRS) pixels of ∼100×100 km, which potentially encompass ecologically heterogeneous regions. Decreasing pixel size would, however, likely lead to the definition of discontinuous biogeographic areas characterized by local ecological conditions rather than broad-scale patterns [21]. Therefore, the use of pixels of 100×100 or 200×200 km has been recommended for delineating biogeographic regions at the continental scale [1].

For some areas, including Germany, UK, Belgium, and Netherlands, information on species distributions per MGRS pixels was readily available from existing atlases, which were resampled at the appropriate resolution level. For all other areas, we retrieved species distribution records from the Global Biodiversity Information Facility (http://www.gbif.org/) when they corresponded to herbarium specimens with precise location information. We further complemented this information by performing a literature search, starting from specialized journals (Journal of Bryology, Arctoa, Cryptogamie, Bryologie, and Nova Hedwigia) and cross-referencing any relevant literature from other documentation sources listed in Table S1. When the same species was reported from the same MGRS pixels more than once, a single presence was retained. The nomenclature was standardized using Grolle & Long [22] for liverworts and Hill *et al*. [23] for mosses, with some modifications to homogenize taxonomic concepts among countries (Appendix S1). The database included 113,321 records for 1,726 species (Table S1), representing 75% of the 453 liverwort, 68% of the 1,292 moss and 73% of the 8 hornwort species of Europe (including Macaronesia).

As opposed to angiosperms, wherein extensive introductions of alien taxa have blurred the biogeographic identity of many areas [24], only 22 cases of introduction and three cases of invasion have been reported in the European bryophyte flora [25]. As a consequence, all of the species were included in the analyses.

### Implementation of Species Distribution Models to Circumvent Data Deficiencies

Species distribution models were used to account for the limitations in data availability (presence of empty MGRS pixels) in some areas. Individual SDMs employing macro-climatic predictors were built from the available presence data. These models were then projected onto the whole area to determine the potential presence of each species in each MGRS pixel from its macro-climatic characteristics. All of those models were subsequently stacked to generate potential species compositions in all pixels.

The distribution range of many bryophyte species spans several continents [10]. We decided, however, to document the niche of the investigated species at the scale of Europe only for two main reasons. First, if a species does not, for whatever reason (e.g., competition), display its complete, potential niche in Europe, we do not feel that it would be useful, for the purpose of describing potential distributions in that continent, to gather information of a potentially wider niche in other continents. Second, documenting the entire niche across the whole distribution range would involve making a major assumption, that is, that the niche is homogeneous across trans-continental distributions. It has long been acknowledged that many bryophyte species exhibit different niches in different biogeographic areas [26], [27]. Furthermore, a growing body of evidence points to high rates of cryptic speciation in bryophytes [28], challenging the notion that continentally disjunct bryophyte populations share a common niche.

The bioclimatic variables available in WorldClim 1.4 (http://www.worldclim.org, [29]) were employed as predictors in the SDMs. In order to obtain identical resolution for climatic variables and species data, we averaged WorldClim data resampled at the 10×10 km scale over each MGRS pixel. Pearson correlation coefficients were computed between each pair of climatic variables over the 1,222 MGRS pixels. To avoid multicollinearity, one of the variables in each pair with a Pearson correlation value >0.8 was eliminated. The variables finally included in the models were bio02 (mean diurnal range), bio04 (temperature seasonality), bio10 (mean temperature of warmest quarter), bio13 (precipitation of wettest month), bio14 (precipitation of warmest quarter) and bio15 (precipitation seasonality).

Species distribution models were constructed with Maxent 3.3.3a [30]. Maxent uses a deterministic algorithm to find an optimal probability distribution based on a set of environmental constraints [31]. It has consistently been shown to be among the highest performing methods [32] and was chosen for this study because (1) it can model species distributions from presence-only species records [30], and (2) its accuracy is not compromised when only a reduced number of records is available [30], [33], [34]. Models were constructed for each of the 1289 species with >10 occurrences in the MGRS pixels to avoid generating low-performance models [34]. The following settings were employed: ‘Auto features’, convergence = 10**^−^** ^5^, maximum number of iterations = 500, regularization value β = 2. Binary models (presence/absence) were generated using the ‘maximum training sensitivity plus specificity’ option implemented in Maxent to avoid overprediction problems [35].

To test the performance of the model independently from the data used to build it, the data were split into a training and a testing dataset including 70% and 30% of the observations, respectively. The performance of the model built from the training set was assessed with the testing set by means of the Area under ROC Curve (AUC) statistic [36]. This operation was repeated ten times and the AUC value averaged over the ten replicates. All the species with an AUC <0.70 were eliminated from subsequent analyses [37], [38].

### Sub-sampling Data to Avoid Distribution Bias

**Figure 1:**
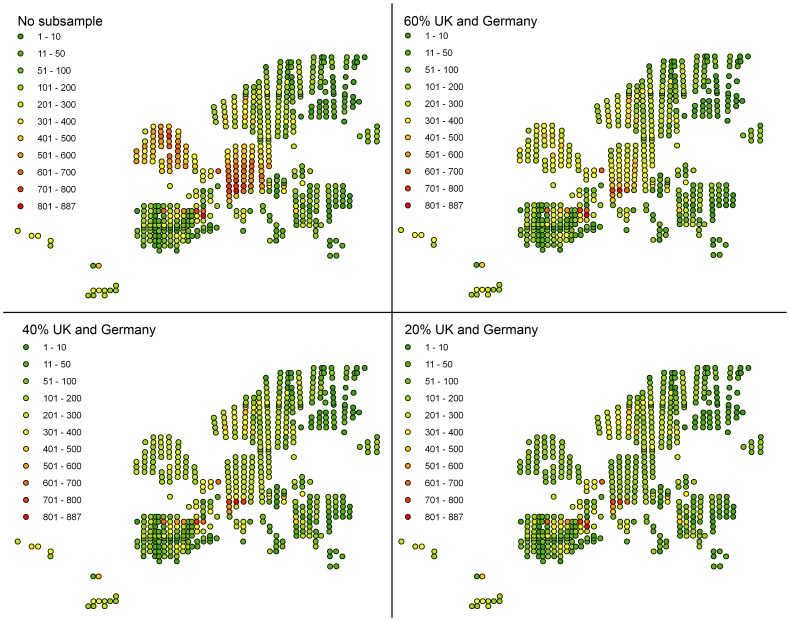
Number of bryophyte species occurrence per MGRS pixel in Europe. when all the data collected (Table S1) are considered (no subsample) and when the most intensively surveyed areas (UK and Germany) are subsampled at 20%, 40% and 60%, respectively.

Species distribution models assume that data for each species represent a random sample of their distribution, since otherwise species niches are biased towards climatic conditions that characterize intensively sampled areas [14]. Given the intensity of records in some areas and their scarcity in others in the present data, this condition is clearly not met (Fig. 1). To circumvent this issue, we randomly sub-sampled data for intensively surveyed areas (Germany and the UK), as recommended by Araújo & Guisan [14] and Hijmans and Elith [20]. Four sub-sampling schemes of those areas (no sub-sample, or 60%, 40% and 20% of the data within Germany and the UK) were applied (Fig. 1), and the analyses re-run in each case to allow subsequent comparison of the impact of the sampling intensity. The total number of species with >10 presences in each datamatrix was 1,258 for no sub-sampling strategy, 1,213 for 60% sub-sampling, 1,181 for 40% sub-sampling and 1,151 for 20% sub-sampling. A further 31 species for the full matrix and 46, 36 and 76 species for the matrices sampled at 60%, 40% and 20%, respectively, were removed from the analyses for exhibiting AUC values <0.70.

### Clustering Analyses

A k-means clustering analysis as implemented by Statistica 8.0 (StatSoft, Tulsa, OK, 2007) was employed to group the MGRS pixels depending on their potential species composition for each of the four sub-sampling schemes. Although the use of several techniques, including UPGMA [1] and eNDeMism [39], has been advocated, k-means was employed here for two main reasons. First, a hierarchical classification was unnecessary and would result in a dendrogram of similarity that would be extremely difficult to interpret with >1000 observations. Second, this technique was most recently employed for other organisms in the same area [5], [6], which allows easy comparisons across taxa.

The analysis was carried out using Hellinger distances in order to avoid taking shared absences into account. For each k values ranging between 2 to 25, 100 iterations were performed and the best run in terms of squared error (sum over the distances of data points from their corresponding cluster centre) was selected. Pixels with <25 predicted species occurrences were found at the eastern margins of the study area. A total of 325 MGRS pixels were discarded because pixels with very few occurrences can bias the results of the k-means analysis by giving strong and misleading signal [6], [16].

Defining the optimal number of clusters (k) to be retained remains an area of controversy [40]. Here, we employed the same v-fold cross-validation procedure as Rueda et al. [5] and Heikinheimo et al. [6] to allow subsequent comparisons of the optimal k value across taxa within the same statistical framework. The v-value for cross-validation was set to 50 and the threshold level of error disparity to 3 and 5%, successively. We represented the clusters obtained for eight k values (3–10) around the optimum k value for the four subsampling intensities, resulting in 32 different biogeographic regionalizations of the European bryophyte flora. We also used geographical contiguity as a criterion to define the optimal classifications obtained [39]. The degree of spatial coherence of the 32 biogeographic regionalizations was measured by Moran’s I autocorrelation coefficient. The latter measures the extent to which pixels assigned to the same region tend to be neighbor from each other or are randomly distributed. A zero value indicates a random spatial pattern, whereas a value of 1 indicates that all the pixels assigned to one region are neighbors from each other.

**Figure 2:**
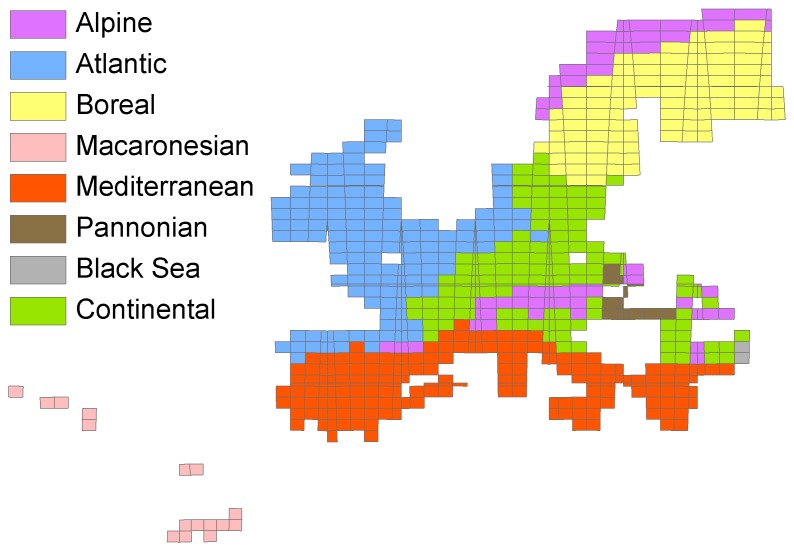
Biogeographical regions of Europe. (http://www.eea.europa.eu/data-and-maps/data/biogeographical-regions-europe-2008) resulting from the ‘Map of Natural Vegetation of the member countries of the European Community and of the Council of Europe’ (Bohn *et al.*, 2000-2004) rescaled at the resolution level of the MGRS pixels.

The biogeographic regionalization selected was compared to the biogeographical regions of Europe (http://www.eea.europa.eu/data-and-maps/data/biogeographical-regions-europe-2008) resulting from the ‘Map of Natural Vegetation of the member countries of the European Community and of the Council of Europe’ [41] (Fig. 2). The similarity of the clusters was assessed by computing the percent of MGRS pixels assigned to one region based upon the bryophyte analyses and to the same region defined by Bohn *et al*. [41]. This overlap does, however, not take into account the percent of MGRS pixels assigned to one region by Bohn *et al*. [41] and to the same region as defined by the bryophyte analyses. We therefore employed a second measure of congruence between the two classifications, namely the kappa statistics [42]. Kappa values were computed with Map Comparison Kit 3.0 (Netherlands Environmental Assessment Agency, http://www.riks.nl/mck/). Following Metzger *et al*. [43], a Kappa value of less than 0.2 represents very poor agreement, 0.2–0.4 poor, 0.4–0.55 fair, 0.55–0.7 good, 0.7–0.85 very good, and greater than 0.85 excellent agreement.

Characteristic species for each of the biogeographic regions obtained were sought by computing species indicator values (IndVal), which range between 0 and 1 and measure both the specificity of a species to a region and its frequency in that region [44]. For each species i in each region j,

Indval = A_ij_ * B_ij_ * 100

where A_ij_ is the number of MGRS pixels of region j where species i was recorded divided by the total number of MGRS pixels where the species was recorded across all regions (specificity), and B_ij_ is the number of MGRS pixels where species i was recorded within region j divided by the total number of MGRS pixels in region j (fidelity).

The biogeographic affinities of each of the indicator species were scored from Düll [45], [46], [47] to produce the biogeographic spectrum of each of the regions resolved.

## Results

**Figure 3:**
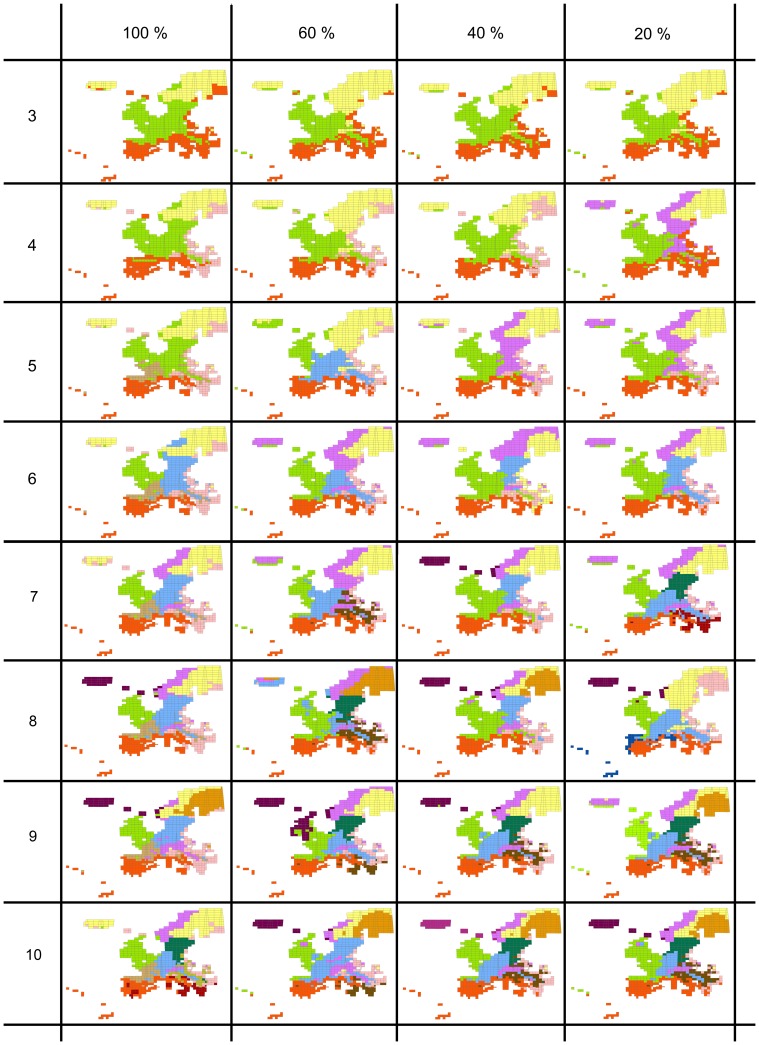
Biogeographic regionalization of the European bryophyte flora inferred from a k-means analysis with k = 3−10. From a datamatrix of potential distribution for each species as inferred from macroclimatic niche modeling. Because of the substantial heterogeneity in floristic sampling, the analyses were performed from models employing the whole range of available data points for each species (no subsampling) and after the random subsampling of 20, 40 and 60% of the data from intensively surveyed areas (UK and Germany) (see Material and Methods).

The optimum k value was 3 when a 5% threshold level of error disparity was applied. When this value was set to 3%, the optimum k was 7 when no subsampling of the intensively surveyed areas was performed, and 5, 5 and 6 for subsampling levels of 20%, 40% and 60% of those areas, respectively. The biogeographic regionalizations of the European bryophyte flora corresponding to and around those optimal k values (k = 3−10) at the different levels of subsampling intensity of the intensively surveyed areas are compared in Fig. 3. Globally, the regions resolved are spatially coherent, as indicated by the comparatively high values of Moran’s I autocorrelation coefficient (all with *p*<0.01) for different values of k and at different subsampling intensities (Table 1). Striking disjunctions were, however, obtained for the lowest and highest values of k (Fig. 3), resulting in the lowest observed Moran’s I statistics.

**Table 1:** Moran’s I autocorrelation coefficient of the k-means clustering of the 100×100 km MGRS pixels across Europe based on their potential bryophyte species composition for different values of k and intensities of subsampling (no subsampling and subsampling at 20, 40 and 60%) of the most intensively prospected areas.

k	S = 20%	S = 40%	S = 60%	S = none
3	0.71	0.73	0.72	0.78
4	0.68	0.72	0.67	0.58
5	0.84	0.66	0.65	0.75
6	0.78	0.73	0.80	0.74
7	0.67	0.82	0.62	0.63
8	0.63	0.79	0.68	0.70
9	0.69	0.68	0.65	0.82
10	0.65	0.60	0.56	0.78

A nested structure emerges for values of k ranging between 3 and 6. For k = 3, Europe is divided into a Mediterranean, Central and Northern region. The proportion of MGRS pixels assigned to the same region in the different analyses based on the complete dataset or datasets subsampled at 20, 40 and 60% of the most intensively surveyed areas is of 83% (see Table S2 in online supplementary material). This indicates that the circumscription of each of those three regions remains largely unaltered when the whole dataset is utilized or when the most intensively surveyed areas are subsampled. The definition of the Mediterranean region remains almost unchanged at higher k values and regardless of subsampling intensity, with an overlap of >90% in the definition of the region at the different subsampling intensities (see Table S1 in online supplementary material).

At k>3, the Central and Northern regions are further split, resulting in a nested structure. At k values of 4 and 5, the overlap in the circumscription of the regions drops to 47.66% and 47.43%, respectively, because the order of split of the central and northern areas differs. Thus for k = 4, the Northern area is split into an Alpine and a Boreal area when the data from the intensively surveyed areas are subsampled at 20%, whereas the Central area is split into a core central area and a marginal, eastern area when the data from the intensively surveyed areas are subsampled at 40%, 60% and 100%. As a consequence, the overlap in the circumscription of the regions is high (>80%) when comparing subsampling intensities of 40%, 60% and 100% but low (about 50%) when comparisons are done at the 20% subsampling intensity. At k = 5, the overlap among biogeographic regions is high (88.31%) at subsampling intensities of 20% and 40%.

At k = 6, a Mediterranean, Atlantic, Continental, Boreal, Alpine and eastern marginal region are resolved. The overlap in the definition of those regions is high (>70%) at the subsampling intensities of 20%, 40% and 60%, but comparisons with the regions defined from the analysis of the full dataset result in overlap ranging between 39.5% to 56%. Indeed, while the main regions (Mediterranean, Atlantic, Continental and Boreal) remain largely unchanged, the Atlantic is further split into a Northern and Southwestern region and the Alpine region is not resolved. Pixels assigned to the eastern marginal region exhibit striking disjunctions into western and northern areas. K-values higher than 7 result in the resolution of areas that become difficult to interpret biogeographically, partly because they progressively exhibit low spatial coherence, as shown by the globally decreasing Moran’s I values (Table 1).

The biogeographic regionalization obtained here with one of the optimal clustering patterns (k = 6 and 60% subsampling), and with one of the highest Moran’s I values (Table 1), was compared to the biogeographical regions of Europe resulting from the ‘Map of Natural Vegetation of the member countries of the European Community and of the Council of Europe’ (Table 2). Apart from an eastern marginal region, which we interpret as an artefact caused by the poor floristic data available for that area (see below), the degree of overlap between the regionalization produced for bryophytes and the biogeographical regions of Europe is of 95% for the Alpine region, 77% for the Atlantic region, 88% for the Boreal region, 88% for the Mediterranean region, and 67% for the Continental region. The global kappa across those regions was 0.709, thus pointing to a very good agreement in the circumscription of the region between the two classifications. The kappa statistics computed for each of those regions ranged between 0.412 (Alpine) and 0.859 (Mediterranean) (Table 2).

**Table 2:** Percentage of MGRS pixels assigned to one region based upon the biogeographic regionalization of European bryophytes (k = 6, 60% subsampling) and to the same region defined in the Biogeographic regions of Europe.

Biogeographic regions(number of MGRS pixels,% of total area)defined for bryophytes	Partitioning of the MGRS clustersassigned to one regiondefined for bryophytes intoregions defined for vegetation	Number of MGRS pixels (%)	kappa
Alpine37 (5.05%)	Alpine	35 (94.59)	0.412
	Atlantic	1 (2.70)	
	Boreal	1 (2.70)	
Atlantic196 (26.78%)	Atlantic	151 (77.04)	0.817
	Continental	31 (15.82)	
	Mediterranean	10 (5.10)	
	Alpine	4 (2.04)	
Boreal119 (16.26%)	Boreal	105 (88.24)	0.807
	Alpine	14 (11.76)	
Continental127 (17.35)	Continental	85 (66.93)	0.568
	Boreal	15 (11.81)	
	Alpine	13 (10.24)	
	Atlantic	11 (8.66)	
	Mediterranean	2 (1.57)	
	Pannonian	1 (0.79)	
Mediterranean170(23.22%)	Mediterranean	150 (88.24)	0.859
	Macaronesian	16 (9.41)	
	Atlantic	2 (1.18)	
	Continental	2 (1.18)	

(http://www.eea.europa.eu/data-and-maps/data/biogeographical-regions-europe-2008, Bohn *et al*. [41]) and kappa statistics.

**Figure 4:**
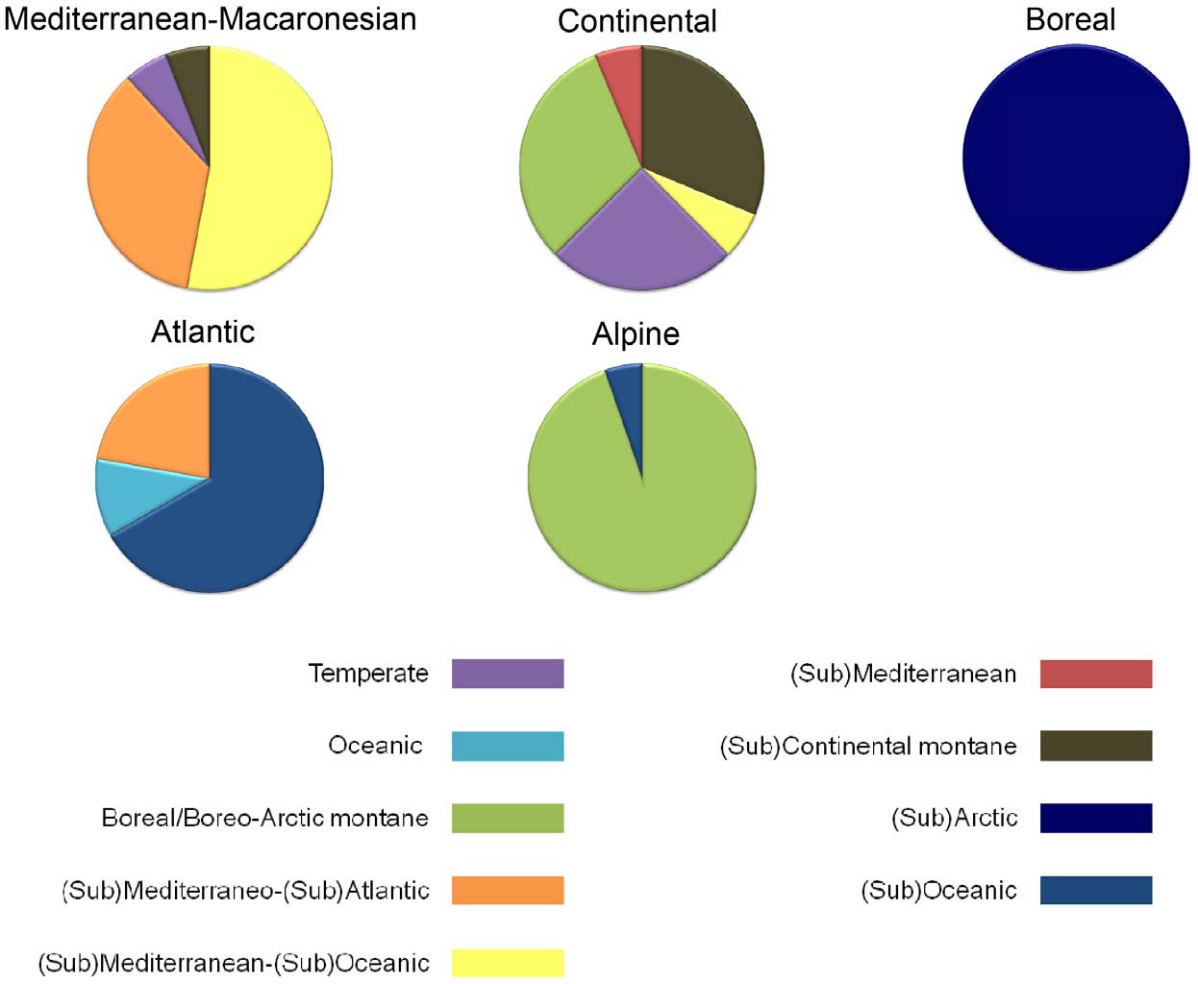
Spectrum of the biogeographical affinities. of the species identified as indicators for the Atlantic, Alpine, Boreal, Continental, and Mediterraneo-Macaronesian regions resolved from the analysis of bryophyte species distributions in Europe.

The twenty species with the highest indicator value for each region (k = 6, 60% subsampling) are provided in Table 3. The regions where the species exhibit the highest indicator values are the Atlantic and Mediterranean (from 0.71 to 0.52 in the Atlantic and from 0.71 to 0.56 in the Mediterranean), whereas the region where the species exhibit the lowest indicator values is the marginal eastern region, where the 20 highest indicator values range between 0.11 and 0.05. The biogeographic spectrum of each of the Atlantic, Alpine, Boreal, Continental, and Mediterraneo-Macaronesian regions based on the biogeographic affinities of their indicator species is presented in Fig. 4. The affinities of the indicator species from the Atlantic, Alpine, Boreal regions are very homogeneous, whereas indicator species for the Mediterranean are of both Mediterranean and Mediterranean-oceanic affinities, and indicator species for the Continental region are very heterogeneous, including a mixture of continental, temperate, and boreal affinities.

**Table 3:** Species indicator value for each of the biogeographic regions defined from the k-means analysis of patterns of potential distribution at the scale of the European bryophyte flora with K = 6 and after the subsampling of the British and German floras at 60% (see Material and Methods for details).

Atlantic		Alpine		Boreal		Continental		MediterraneanMacaronesian	
*Porella pinnata*	0.71	*Didymodon* *icmadophilus*	0.57	*Splachnum rubrum*	0.66	*Mnium spinulosum*	0.54	*Barbula ehrembergii*	0.71
*Saccogyna viticulosa*	0.66	*Herzogiella striatella*	0.53	*Sphagnum wulfianum*	0.64	*Rhodobryum ontariense*	0.5	*Entosthodon convexus*	0.7
*Andreaea mutabilis*	0.64	*Bryhnia scabrida*	0.5	*Warnstorfia tundrae*	0.63	*Rhynchostegium rotundifolium*	0.48	*Campylostelium pitardii*	0.68
*Lejeunea lamacerina*	0.61	*Bryum salinum*	0.49	*Calliergon megalophyllum*	0.62	*Tortella bambergeri*	0.46	*Rhynchostegiella litorea*	0.66
*Didymodon sinuosus*	0.59	*Cynodontium jenneri*	0.49	*Dicranum drummondii*	0.62	*Sciuro-hypnum flotowianum*	0.45	*Grimmia pitardii*	0.65
*Hyocomium armoricum*	0.59	*Dicranum leioneuron*	0.49	*Hamatocaulis lapponicus*	0.62	*Mnium lycopodioides*	0.44	*Barbula bolleana*	0.63
*Orthotrichum sprucei*	0.59	*Bryum neodamense*	0.48	*Calliergon richardsonii*	0.61	*Bryum schleicheri*	0.43	*Octodiceras fontanum*	0.63
*Fissidens celticus*	0.56	*Cynodontium fallax*	0.47	*Racomitrium microcarpon*	0.6	*Brachythecium geheebii*	0.42	*Scorpiurium deflexifolium*	0.62
*Fissidens monguillonii*	0.56	*Grimmia incurva*	0.46	*Loeskypnum badium*	0.59	*Cleistocarpidium palustre*	0.42	*Timmia bavarica*	0.62
*Barbilophozia atlantica*	0.55	*Hygrohypnum alpinum*	0.46	*Sphagnum jensenii*	0.59	*Fissidens gracilifolius*	0.42	*Phymatoceros bulbiculosus*	0.61
*Plagiochila spinulosa*	0.55	*Kiaeria falcata*	0.46	*Splachnum luteum*	0.59	*Hypnum vaucheri*	0.42	*Cheilothela chloropus*	0.6
*Scapania glaucocephala*	0.55	*Sciuro-hypnum glaciale*	0.46	*Warnstorfia trichophylla*	0.59	*Lophozia wenzelii*	0.42	*Corsinia coriandrina*	0.6
*Scleropodium caespitans*	0.55	*Cynodontium gracilescens*	0.45	*Cnestrum schisti*	0.58	*Brachythecium tommasinii*	0.41	*Homalothecium aureum*	0.6
*Calypogeia arguta*	0.54	*Hygrohypnum smithii*	0.45	*Neckera oligocarpa*	0.58	*Cinclidotus riparius*	0.41	*Bartramia stricta*	0.59
*Fissidens crispus*	0.54	*Kiaeria blyttii*	0.45	*Fontinalis dalecarlica*	0.56	*Dicranum viride*	0.41	*Didymodon sicculus*	0.59
*Marchesinia mackaii*	0.54	*Paraleucobryum enerve*	0.45	*Warnstorfia procera*	0.56	*Fissidens gymnandrus*	0.4	*Orthotrichum acuminatum*	0.57
*Riccia subbifurca*	0.54	*Plagiothecium piliferum*	0.45	*Pogonatum dentatum*	0.55	*Timmia bavarica*	0.4	*Pottia intermedia*	0.57
*Kurzia sylvatica*	0.53	*Syntrichia norvegica*	0.45	*Sphagnum subfulvum*	0.55	*Orthotrichum patens*	0.39	*Entosthodon durieui*	0.56
*Orthotrichum rivulare*	0.53	*Tayloria tenuis*	0.45	*Drepanocladus sordidus*	0.53	*Physcomitrium eurystomum*	0.39	*Riccia macrocarpa*	0.56
*Heterocladium flaccidum*	0.52	*Andreaea frigida*	0.44	*Ulota curvifolia*	0.51	*Scapania calcicola*	0.38	*Scorpiurium circinatum*	0.56

Only the 20 species with the highest indicator values are given for each region.

## Discussion

### Modeling Species Distributions for Biogeographic Regionalization

Species distribution models were produced at the scale of the entire bryophyte flora of Europe to overcome the limited amount of distributional information in many areas with the aim of achieving the first biogeographic regionalization for this group. This approach, which could potentially be implemented in other taxa with limited distributional information, assumes that macroclimatic suitability reflects actual distributions. Species distributions are, however, potentially influenced by a range of factors operating over a range of temporal and spatial scales and, in particular, dispersal limitation and historical factors that are difficult to include in species distribution models (but see [48]). Consequently, discrepancies between potential and actual species distributions point to sometimes substantial niche unfilling owing to dispersal constraints on post-glacial expansion [49]. For example, Normand *et al.* [50] showed that postglacial migration was the strongest determinant for one-sixth of the 1,016 European plant species investigated, in particular for species with limited long-distance dispersal ability. As a result, high levels of compositional error (mean of 60% relative to the observed values) were reported in certain studies attempting to assess the performance of species distributions models at a fine resolution and local scale [51].

Two features of the present study suggest, however, that the species distribution models produced here for the entire European bryophyte flora provide a good approximation of the actual species distributions. First, error rates between predicted and observed species assemblages per pixel drastically diminish when model predictions are examined over large study areas relative to species range sizes [51]. One possible reason for this is that, at large geographical scales, climate exerts the dominant control on species distributions [52]. This is perhaps even truer in bryophytes whose poikilohydric condition implies that they obtain water directly from rainfall and can resume physiological activity only in the wet state [53]. As a consequence, macroclimatic factors proved excellent predictors of bryophyte-dominated ecosystems and bryophyte species distributions [54].

Second, bryophytes are typically perceived as extremely efficient dispersers [7], diminishing the concern about niche unfilling owing to dispersal constraints. In fact, fast and massive post-glacial migrations of bryophytes in Europe have been shown by phylogeographic analyses. In the genus *Polytrichum* for instance, van der Velde & Bijlsma [55] found no geographic structure in patterns of genetic variation across the continent. In *Sphagnum fimbriatum* and *Radula lindenbergiana*, the dominance of one haplotype suggests rapid post-glacial dispersal, sufficient to prevent substantial genetic differentiation among populations by genetic drift and to wipe out an initially present genetic structure resulting from the last glacial periods [56], [57]. This is consistent with previous studies reporting rapid community shifts from range dynamics analyses [58], [59] and stratigraphic analyses of macro-remains preserved in peat [60], [61]. The potential of bryophytes for a high efficiency to colonise habitats as soon as they become available suggests that the time-lag between habitat availability and colonization is reduced, thereby making it possible to use maps of ecological suitability as proxies for actual distributions.

The use of SDMs to overcome the limited amount of distributional data in biogeographic regionalization may also be potentially limited by the number of species for which reliable SDMs can be constructed. Here, we discarded all of the species with fewer than 10 occurrences and an AUC<0.70 in the cluster analyses, which represented 34 to 42%, depending on the datasets used, of the total number of species initially included. Species with low predictabilities are precisely those either exhibiting the highest dispersal capacities and widest ecological ranges, or narrow endemics [62], [63], which typically are unlikely to contribute to the biogeographic patterning at the continental scale. Hence, accurate biogeographic regionalisations can be achieved through the analysis of a reduced set of species with informative distribution patterns [64].

Finally, the SDM approach employed here is sensitive to sample size and biases in the distribution of data [14], making it necessary to assess the impact of different subsampling schemes of the most intensively surveyed areas. Several discrepancies were observed, especially between the full and subsampled datasets, with for instance the resolution of a northern and southern Atlantic region and the absence of the Alpine region in the former case. There is no objective criterion to determine which of the different subsampling schemes is the most appropriate. Because of the failure of the analysis based on the full dataset to resolve the Alpine region, which is arguably a major biogeographic entity at the European scale [65], we favour the subsampling approach. Globally, however, congruent results were obtained in terms of geographic regionalizations using different subsampling schemes of the intensively surveyed areas, suggesting that a strong and dominant signal was present in the data.

### Biogeographic Regionalization of the European Bryophyte Flora

The congruence of the clusters resolved here from the analysis of bryophyte species distributions with large-scale vegetation patterns [41] is at odds with our primary hypothesis that substantial differences in ecophysiology and distributions between vascular plants and bryophytes would result in different biogeographic patterns. The first divisions (k = 2−4) involve a North/South differentiation into Mediterranean, Central, and Northern regions that closely match those observed for the analysis of vascular plant species [6]. From k = 5, a western versus eastern disjunction is resolved. The degree of spatial overlap between the continental regions resolved by the bryophytes and vascular plants is only of about 67%. In fact, the Continental region defined by bryophyte species distributions is weakly characterized by a mixture of species with different biogeographic affinities and not, as is the case of all other regions, by specific suites of characteristic species. Another eastern continental region is resolved. It exhibited the lowest species indicator values, suggesting that this region is negatively characterized by species absences and not by the presence of key taxa. This points to an artefact due to the poor floristic records available for the area and suggests that this region should be ignored.

To the West, the Atlantic region resolved here perfectly corresponds to the one that has been identified in previous phytogeographic classifications [66]. As opposed to previous phytogeographic treatments that included the western side of Scandinavia and the Iberian Peninsula to the Atlantic region based on their vascular plant assemblages [66], [67], the present analyses support recent evidence that the Atlantic region should be restricted to the British Isles, the western side of France and Benelux [6]. The presence of such hyper-oceanic species as *Saccogyna viticulosa* and *Dicranum scottianum* in western Scandinavia and western Iberian Peninsula should hence be considered as an irradiation of an Atlantic element into regions that otherwise belong to the core Alpine and Mediterranean regions, respectively. This spatial congruence found between biogeographic regions defined for vascular plants and bryophytes reinforces the notion that, despite the substantial differences in ecophysiology and dispersal ability between the two groups, post-glacial migration patterns might have been much more similar between them than previously thought [68].

A signature of bryophyte’s high dispersal ability in the regionalization obtained can, however, be found at two levels. First, the optimal number of clusters retained in the present analyses was 3−6 (or 5 without the spurious eastern continental region, see above) depending on the threshold level of error disparity and the sub-sampling strategy employed. This number is substantially lower than the optimal k value of 12 obtained for the analysis of vascular plant distributions using exactly the same statistical procedure [6]. Such differences in the number of regions retained within an explicit statistical framework using the same optimality criterion for the numbers of clusters to be retained have been interpreted in terms of differences in dispersal capacities [5]. The differences observed between the optimal number of regions retained from the analyses of bryophyte and vascular plant distributions hence support our second hypothesis that analyses of bryophyte species distributions would result in lower regions than analyses of vascular plants. In other words, wide-scale distribution patterns of bryophyte species would preclude the recognition of smaller, nested biogeographic regions revealed by analyses of taxa with lower mobility, and hence, higher ‘degree of resolution’.

The higher resolution level of vascular plant as compared to bryophytes in biogeographic regionalization is best exemplified by the Mediterranean. In the analyses of Heikinheimo *et al*. [6], the Mediterranean cluster is separated at the earliest stage of the procedure as a single area, which corresponds to the Mediterranean area of Bohn *et al.* [41]. The Mediterranean area is readily identified as a well-circumscribed region in both bryophyte and vascular plant analyses by species that are specific to it and are largely distributed across the region. Fossil and phylogeographic evidence suggests that these species managed to migrate efficiently across the region during the last glacial/interglacial cycles, while their restriction to the current Mediterranean is due to the reduction of a formerly wider, extra-Mediterranean range during the last glacial maximum [69], [70]. In vascular plants, the presence of narrow endemics [71] with limited dispersal capacities [50], [72], [73] further allows the resolution of finer geographical entities, with five nested clusters within the Mediterranean mirroring the floristic heterogeneity of the Mediterranean area [6]. In contrast, bryophytes' rates of endemicity within the Mediterranean are virtually zero [74], precluding the emergence of a nested pattern.

More surprisingly, the same considerations also apply to the Atlantic region. While the circumscription of the region as a whole is remarkably similar between bryophytes and vascular plants, analyses of the latter further resolved a nested hyper-oceanic element [6], [75]. Despite the presence of a suite of species with hyper-oceanic affinities that indeed belong to genera otherwise tropical in distribution (e.g., *Leptoscyphus*, *Dumortiera*, [13]), such a hyper-oceanic element was not resolved in the present analyses. This failure may either result from the coarse grain size employed in the present study or from the negligible contribution of those hyper-Atlantic species as compared to the bulk of more widely distributed Atlantic species present in those areas.

A second signature of the high dispersal ability of bryophytes in their biogeographic patterns is that, as opposed to the traditional view of Macaronesia as a distinct biogeographic region [41], the analyses presented here failed to resolve a Macaronesian element. While the angiosperm flora of the Canaries and the Azores is indeed comprised of >40% endemics that contribute to the recognition of a Macaronesian region, the extremely low levels of endemicity in the bryophyte flora (<2%), which has been interpreted of either intense gene flow preventing endemic speciation [76] and/or fast rates of continental colonization [77], preclude the characterization of a Macaronesian element for bryophytes. The resolution of a heterogeneous Macaronesian region in some analyses, with the Canaries assigned to the Mediterranean region, whereas Madeira and the Azores are assigned to the Atlantic region, points to the dynamic interchange between those respective areas, precluding the characterization of a coherent Macaronesian region [78].

### Conclusion

Limitations in the availability of species distribution data are a general issue in taxa that are less studied than vertebrates or seed plants, as well as in many tropical areas. Despite its importance, integration of information across existing biodiversity monitoring schemes remains poor [79]. Even in Europe, only 23 out of 547 monitoring schemes assemble data at an international or continental level [79], [80]. We attempted to circumvent this issue in the European bryophyte flora by implementing predictive distribution models based on macroclimatic predictors. This approach allowed us to explore general trends of macro-ecological species assemblages, but is based on many assumptions that can only be tested when additional data on species distributions become available. This highlights the substantial importance of developing integrated mapping projects of bryophyte, and other overlooked taxa, in key biogeographic areas of Europe, and the Mediterranean peninsulas in particular.

## Supporting Information

Table S1 **List of literature references used for the datamatrix.**
(DOC)

Table S2 **Overlap (percent of MGRS pixels assigned to one region based upon the bryophyte analyses and to the same region defined by Bohn et al.** [**41**] **) in the definition of the biogeographic regions based upon the similarity in their bryophyte species assemblages between different subsampling strategies of the most intensively surveyed areas.** (S00, S20, S40, S60 corresponding to “no subsample” and subsample at an intensity of 20%, 40%, and 60%, respectively) for values of k ranging between 3 to 6.
(XLS)

Appendix S1 **Nomenclature followed for moss and liverwort species with corresponding references.**
(DOC)
