# Supplementary material for: Modeling Species Distributions from Heterogeneous Data for the Biogeographic Regionalization of the European Bryophyte Flora
Source: PLoS One. 2013 Feb 11;8(2):e55648. doi: 10.1371/journal.pone.0055648 (PMC3569459; doi:10.1371/journal.pone.0055648)
Supplement: Table S1 — List of literature references used for the datamatrix. (DOC) [file pone.0055648.s001.doc]

TABLE S1. List of references used to compile the dataset and table with the corresponding MGRS squares.

1 Albertos, D., Lara, F., Garilleti, R. & Mazimpaka, V. (1997) Estudio briofloristico de una formacion relictica de Prunus lusitanica L. de la Sierra de Gredos (Avila, Espana), *Cryptogamie, Bryologie, Lichenologie*, 18, 4, 303-313.

2 Aleffi, M., Sabovljević & Tacchi, R. (2004) Bryoflora of Monte Conero Regional Park (Marche, Italy), *Flora Mediterranea* , 14.

3 Aleffi, M. (2005) New checklist of the Hepaticeae and Anthocerotheae of Italy, *Flora Mediterranea*, 15, 484-566.

4 Aleffi, M. & Esposito, A. (2005) The bryoflora of Castelvolturno Nature reserve (South Italy) as indicator of environnemental factors of disturbance, *Flora Mediterranea*, 15, 669-682.

5 Aleffi, M., Barbo, M., Bertani, G., Bonini, I., Buffa, G., Carratello, A., Cogoni, A., Flore, F., Miserere, L., Perego, S., Piovano, S., Puntillo, D., Schumaker, R. & Sguazzin, F. (2003) Contributo alla conoscenza briologica della valle Stura di Demonte ( Alpi Occidentali, Piemonte, Italia), *Bollettino Regione Piemonte*, vol. 21, n°1, 227-248.

6 Aleffi, M., Schumacker, R., Puntillo, D., Privatera, M., Bernardo, L. & Raso, C. (1998) Dumortiera hirsuta (sw.) Nees in Calabria Italy, Its southern most and highest localities in Europe, and addition to the bryophyte flora of this region, *Cryptogamie, Bryology, Lichenology*, 19, 4, 341-359.

7 Anales de Biología 26: 117-155, 2004,Bryophyte Check-list of Murcia Province (Southeastern Spain),María J. Cano, Juan A. Jiménez, M. Teresa Gallego, Rosa M. Ros & Juan Guerra,Departamento de Biología Vegetal, Área de Botánica, Facultad de Biología, Universidad de Murcia, Campus de Espinardo, 30100 Murcia, Spain.

8 Armata , L. (2005) A contribution to the bryoflora of the Pogorze Dynowskie Foothills (Western Carpathians), *Annales Universitatis Mariae Curie-Sklodowska Lublin – Polonia*, Sect. 3, Biologia, Vol. 60.

9 Armata , L. (2006) New records of rare and endangered mosses from the Bieszczady Zachodnie Range and the Carpathian Foothills, *Annales Universitatis Mariae Curie-Sklodowska Lublin – Polonia* Sect. 3, Biologia. Vol. 61.

10 Armata, L. (2008) A contribution to the moss flora of the eastern part of the Polish Carpathians, 169–178, in Stebel, A. & Ochyra, R., *Bryophytes of the Polish Carpathians*. Sorus. Poznań, Poland, 160pp, ISBN 83-87133-71-X

11 Armata, L. & Zubel, R. (2007) Bryophyte flora of the "Gory Pieprzowe" Nature Reserve on Wyzyna Sandomierska, SE Poland, *Annales Universitatis Mariae Curie-Sklodowska Lublin – Polonia* Sect. 3, Biologia, Vol. 62.

12 Banc de Dades de la Biodiversitat de Catalunya: http://biodiver.bio.ub.es/biocat/homepage.html

13 Bardat, J. (1997) La flore bryophytique de la forêt de Rambouillet (France), région Ile-de-France, Département des Yvelines, *Cryptogamie, Bryologie, Lichénologie*, 18, 2, 87-120.

14 Bardat, J. & Hugonnot, V. (2002) Apercu de la flore et de la végétation bryophytique du site des Narces d'Isslanlas (Ardèche, France), Témoin exceptionnel d'une zone humide de moyenne montagne, *Cryptogamie, Bryologie*, vol 23, 1, 51-72.

15 Bates, J.W.(1991) Brioflora of Belle-ile Brittany and comparison with the Channel island, *Cryptogamie, Bryologie, Lichenologie*, 12, 2, 111-148.

16 Bischler, H. & Jovet-Ast, S. (1979) Nouvelles récoltes d'hépatiques en Crête, *Revue Bryologique et Lichenologique*, 45, 1, 45-60.

17 Bischler, H., Jovet-ast, S. & Baudouin, R. (1980) Hépatiques de la côte Albanaise, *Cryptogamie, Bryologie, Lichenologie*, 1, 3, 247-267.

18 Blockeel, T.L., Ros, R.M., Sabovljevic, M., Cano, M. J., Galego, M.T. & Muñoz, J. (2002) New and interesting bryophytes records for Greece, *Cryptogamie, Bryologie*, 23, 2, 149-155.

19 Bonnini, I., Casini, F., Chiarucci, A. & De Dominicis, V. (2005) The bryophyte flora of the geothermal fields of Sasso Pisano (Pisa, Italy), *Cryptogamie, Bryologie*, 26, 3, 291-299.

20 Borges P.A.V., Cunha, R., Gabriel, R., Martins, A.F., Silva, L. & Vieira, V. (2005) A list of the terrestrial fauna (Mollusca and Arthropoda) and flora (Bryophyta, Pteridophyta and Spermatophyta) from the Azores, Direcçao Regional do Ambiente and Universidade dos Açores, Horta, Angra do Heroismo and Ponta Delgada, Portugal.

21 Brugués, M., Cros, R.M. & Sérgio, C. (2010*) Cartografia de Briòfits. Península Ibèrica i Illes Balears. Recurs electrònic http://briofits.iec.cat * Data d'actualització del mapa

22 Buffa, G. & Miserere, L. (2004) La brioflora delle rupi serpentinitiche e gneissiche (Parco Naturale Regionale del M. Avic, Parco Nazionale del Gran Paradiso). *Revue Valdôtaine d’Histoire Naturelle*, 58, 7-19.

23 Burgaz, A.R. & Fuertes, E. (1992) Apportacionnes a la vegetacion epifita (briofitos y liquenes).II. (La Rioja, España), *Cryptogamie, Bryologie, Lichenologie*, 13, 2, 133-153.

24 Casas, C., Burgués, M., Cros, R.M., Sergio, C. (1996) Bryophyte Cartography of the Iberian Peninsula, Balearic and Canary islands, Azores and Madeira, fascicules I, II, III,IV, Barcelona, Institut d'estudis catalans.

25 Celle, J. (2008) Atlas préliminaire des bryophytes de la Haute-Garonne, ed. Isatis 31, Pouze, Supplément au n° 8., 3-153, ISSN 1632-5826.

26 Cipollaro, S. & Colacino, C. (2005) Flora of the beech silver fir coenosis of Mount Motola (National Park of Cilento and Vallo di Diano) Teggiano ( Salerno, S-Italy), *Flora Mediterranea*, 15, 385-395.

27 Cogoni, A., Flore, F. & Aleffi, M. (2002) Survey of the bryoflora on Monte Limbara (Northern Sardinia), *Cryptogamie, Bryologie*, vol.23, 1, 73-86.

28 Cogoni, A., Flore, F. & Scrugli, A. (2004) The bryological flora of Isola dei Cavoli ( S-E Sardinia, Italy), *Flora Mediterranea*, vol 14, 115-127.

29 Cogoni, A., Flore, F., Adamo, C. & Scrugli, A. (2007) The bryophytic flora of the Molara Island (Northeastern Sardinia), *Flora Mediterranea*, 17, 185-204.

30 Colacino, C. & Sabovljević, M. (2006) Bryophyte flora of Albania: a preliminary checklist, *Cryptogamie, Bryologie*, 27, 4, 1-30.

31 Cortini-Pedroti, C. (1985) La florule bryologique des collines sabloneuses à l'ouest du lac Trasimène (Ombrie), *Cryptogamie, Bryologie, Lichenologie*, 6, 1, 59-63.

32 Cortini-Pedrotti, C. (2010) Checklist of Italian mosses, Dipartimento di botanica ed Ecologia, Universita di Camerino, Italia : http://dbiodbs.univ.trieste.it/global/mosses1

33 Cvetić, T. & Sabovljević, M. (2004) New and interesting bryophyte records for Montenegro, *Phytologia Balcanica*, 10, 2-3, 67-69.

34 Database compiled for the Flora Briofitica Iberica project (grant PB96-IIII-CO2 of the Ministry of Education and Science of Spain)

35 Database of the Bryophyte Collection of HNHM ; Hungary, Bükk Mts, Aggtelek National Park, surrounding of Salgótarján ; Slovakia, Slovakian karst.

36 Database of the Bryophyte Collection of HNHM; Hungary, Kőszegi, Soproni Mts, Ásványráró, surrounding of Ajka, Fertő lake, Vasvár.

37 Database of the Bryophyte Collection of HNHM; Hungary, Tapolcai basin, Szent György hill, Badacsony hill, Keszthelyi Mts, Őrség, other data from Zala county. [with addition for Pohlia from Erzberger, P. (2005) The bulbilliferous species of Pohlia (Bryaceae, Musci) Studia Botanica Hungarica, 36, 67-75. - for Schistidium from Erzberger, P. & Schröder, W. (2008) The genus Schistidium (Grimmiaceae, Musci) *Studia Botanica Hungarica*, 39, 27-88. -for Grimmia from Erzberger, P. (2009) The genus Grimmia and Coscinodon (Grimmiaceae, Musci) *Studia Botanica Hungarica*, 40, 37-124.]

38 Dhien, R. (1977) La végétation bryologique des stations thermales II. La source Saint-Aré à Décize (Nièvre), *Revue Bryologique et Lichenologique*, 43, 3, 353-355.

39 Dhien, R. (1980) Florule bryologique des ruines, Cryptologie ,bryologie , lichenologie, 1, 3, 315-316.

40 Dirkse, G.M. & Losada-Lima, A. (2011) Additions and amendments to the moss flora of the Canary Islands, *Cryptogamie, Bryologie* 32, 1, 37-41

41 Dolnik, C. & Napreenko, M.G. 52007) The bryophytes of the Southern Curonian split, *Arctoa*, 16, 35-46.

42 Dragicevic, S., Veljic, M. & Marin, P.D. (2008) Liverworts from the Moraca river basin-Taxonomic and ecological analysis, *Archives of Biological Science Belgrade*, 60, 3, 469-474.

43 Ederra Indurain, A. (1988) Briofites del Pirineo de Navarra (España), *Cryptogamie, Bryologie, Lichenologie*, 9, 2, 103-107.

44 Frahm, J.P. (2002) Bryoflore des Vosges et des zones limitrophes, *Limprichtia*, 19, 322pp., ISSN 0943-8823.

45 Fuertes Lasala, E. & Alvarez Orzanco, J. (1982) Aportaciones a la flora briologica espanola: El Señorio de Bertiz (Navarra), *Cryptogamie, Bryologie*, 3, 2, 139-155.

46 Fuertes Lasala, E. & Martinez- Condé, E. (1988) Vegetacion briofitica del macizo oriental de los Picos de Europa (Andarra and Cantabria), España, I. Communidades saxicolas, acuaticas y subacuaticas, *Cryptogamie, Bryologie, Lichenologie*, 9, 2, 109-127.

47 Fuertes Lasala, E. & Martinez- Condé, E. (1989) Addition to the bryoflora of the picos de Europa, of Cantabria and the Iberian peninsula, *Cryptogamie, Bryologie, Lichenologie*, 10, 4, 319-324.

48 Ganeva, A. & Natcheva R. (2003) Checklist of the bryophytes of Bulgaria with data on their distribution. I. Hepaticae and Anthocerotae, *Cryptogamie, Bryologie*, 24, 3, 229-239.

49 Ganeva, A. & Natcheva, R. (2003) Checklist of the bryophytes of Bulgaria with data on their distribution. I. Hepaticae and Anthocerotae, *Cryptogamie, Bryologie*, 24, 3, 229-239.

50 Garcia Gomez, R. & Fuertes Lasala, E. (1980) Communidades mucinales de los Saladares y Espartales de Navarra ( España), *Cryptogamie, Bryologie , Lichenologie* , 1, 3, 289-304.

51 Gil, J.A., Guerra, J. (1985) Estudio briosociologico a la Sierra de la Demanda y Urbion (Espana), *Cryptogamie, Bryologie, Lichenologie*, 6, 3, 219-258.

52 Gimeno-Colera, C. & Puche-Pinazo, F. (1999) Flora y vegetacion briofitica higro-hidrofila de la communidad Valenciana (Este de España), *Cryptogamie, Bryologie*, 20, 1, 49-68.

53 Gonzalez-Mancebo, J.M., Francisco Romaguera, F., ROS, R.M., Jairo Patino, J. & Werner, O. (2008) Bryophyte flora of the Canary Islands : an updated compilation of the species list with an analysis of distribution patterns in the context of the Macaronesian Region, *Cryptogamie, Bryologie*, 29, 315-357.

54 Hans, F. (1998) Die Moosflora der kleinen Luxemburger Schweiz (Mullertal) von Luxembourg, Ministère de la culture travaux scientifiques du Musée National d'Histoire Naturelle de Luxembourg, vol.28, ISSN 0251-2424.

55 Hauguel, J.C. & Wattez, J.R. (2008) Iventaire des bryophytes de Picardie: présence, rareté et menace octobre, Conservatoire Botanique National de Bailleul, Rapport d’étude, 38pp.

56 Hébrard J.P. (2006), Contribution à l'étude bryologique du Petit Lubéron (Vaucluse II), La crête, le versant sud et l'extrémité occidentale du massif, *Cryptogamie Bryologie*, 27, 2, 253-264.

57 Hebrard, J.P. (1980) Contribution à l'étude des Muscinées du Parc naturel des Ecrins. Observations floristiques et écologiques, *Cryptogamie, Bryologie, Lichenologie*, 1, 4, 339-397.

58 Hébrard, J.P. (1990) Contribution à l'étude bryologique du Petit Lubéron (Vaucluse I), Les contreforts orientaux (région de Bonnieux), Cryptogamie, Bryologie-Lichenologie, 11,3, 319-328.

59 Herzberger, P. Contribution to the bryophyte flora of Chalkidikki, Greece, *Willdowia*, 36, 2006, 515-525.

60 Hill, M.O., Preston, C.D., Smith, A.J.E. (1991) Atlas of the bryophytes of Britain and Ireland: I Liverworts, II & III Mosses, Harley Books, 352pp

61 Huguennot, V. (2010) Mousses et hépatiques de Païolive (Ardèche et Gard, France), *Bulletin de la société botanique du centre-ouest*, numéro spécial, 34, 293pp.

62 Infante Sànchez, M. (2000) Las hepaticas y antocerotas (marchantiophyta y anthocerotophyta) en la communidad autonoma del pais vasco, universitad del pais vasco, euskal herriko unibertsitatae, vol 6, Guineana.

63 Infante Sànchez, M. & Heras Pérez, P. (2005) Bryophytes des étages subalpin et alpin de Barroude et Port Vieux (Aure, Parc National des Pyrénées, France), *Bulletin de la Société d’Histoire Naturelle de Toulouse*, 141, 2, 103-108.

64 Infante Sanchez, M. & Heras Perez, P. (2007) Briofitos (Musgos y Hepaticas del Parque Natural de la Sierra y los Cañones de Guara, colleccio pius font i quer, vol.5, Institut d'estudis Ilberdencs, Disputacio de Lleida, 236pp., ISBN 8489943982 9788489943988.

65 Infante Sànchez, M. & Heras Pérez, P. (2007) Briofitos (musgos y hepaticas) del Parque natural de la Sierra y los Cañones de Guara, institut destudis ilerdencs, 236pp., ISBN 9788489943988 8489943982.

66 Jakovljev, Z., Sabovljević, M., Zechmeister, H. (2009) Contribution to the bryophyte flora of Donau-Auen National Park (Austria), *Folia Cryptogamica Estonica*, 45, 49–54.

67 Kurschner, H. & Parolly, G. (1997) Addition to the bryophyte flora of the Durmitor National Park (Crna-Gora) and the first conspectus of all records, *Willdenowia*, 27, 249-255.

68 Lara, F. & Mazimpaka, V. (1994) Briofitos corticolas de los robledales de la sierra de Gredos (Avila), España, Cryptogamie, Bryologie, *Lichenologie*, 15, 2, 161-169.

69 Lara, F., Lopez, C. & Mazimpaka, V. (1991) Ecologia de los briofitos urbanos en la ciudad de Segovia (España), *Cryptogamie, Bryologie, Lichenologie*, 12, 4, 425-439.

70 Lloret, F., Cros, R.M., Brugues M., & Granzow de la Cerda, I. (1997) Aspectos biogeographicos y chorologicos de los briofitos de la Sierra de Gredos, Espana, *Cryptogamie Bryologie Lichenologie,* 18, 2, 151-164.

71 Lo Giudice, R. & Cristaudo, A. (2004) Chorological and ecological survey on the vascular and bryophitic flora in Enna territory (Erei mountains, C-Sicily), *Flora Mediterranea*, 14, 357- 417.

72 Luna, J. & Estebanez,B. (2008) Brioflora de Valsain (Segovia): Catalogo y observaciones chorologicas, Boletin de la Societad Espanola de Briologia, 32-33, 9-19.

73 Brugues, M. (1978) Flora briologica de los estratos des buntsandstein de la Cordillera Costero Catalana, *Cryptogamie Bryologie Lichenologie,* 44, 2, 149-201.

74 Martincic, A. (2009) Contribution to the bryophyte flora of Republic of Macedonia, *Hacquetia*, 8, 2, 97-114.

75 Martinez-Abaigar, J. & Ederra, A. (1992) Brioflora del Rio Iregua (La Rioja), España, *Cryptogamie, Bryologie, Lichenologie*, 13, 1, 47-69.

76 Martinéz-Abaigar, J., Nuñez-Olivera, E. & Beaucourt, N. (2002) Moss communities in the irrigation channels of the river Irigua basin, (La Rioja, Northern, Spain), *Cryptogamie, Bryologie*, 23, 2, 97-117.

77 Medina, N.G., Medina, R., Lara, F. & Mazimpaca, V. (2008) Brioflora epifita de Sierra Alhamilla (Almeria), *Boletin de la Societad Espanola de Briologia*, 32-33, 1-7.

78 Meinunger, L. & W. Schröder (2007) Verbreitungsatlas der Moose Deutschlands. Herausgegeben von O. Dürhammer für die Regensburgische Botanische Gesellschaft von 1790 e. V., 2044 pp., Regensburg.

79 Moya, J.J., Ros, R.M. & Guerra, J. (1994) Bryophyte flora and vegetation of the flora del Maigmo, Alicante, Spain, *Crytogamie, Bryologie, Lichenologie*, 15, 1, 43-65.

80 Natcheva, R. (2010) New bryophyte records in the Balkans: 6, *Phytologia Balcanica* 16, 1, 137-141.

81 NISM (2004) Online-Atlas of Swiss Bryophytes. - http://www.nism.uzh.ch (« 2011 »)

82 Notov, A.A., Spirina, U.N., Ignatova, E.A., Ignatov, M.S. (2002) Moss of the Tver province, Middle part of European Russia , *Arctoa* ,11, 297-332.

83 Olivan, G., Fuertes, E. & Acon, M. (2001) Flora bryologica de la Sierra de Pennia Sagra (Cantabria Espana), *Cryptogamie, Bryology*, 22, 2, 129-144.

84 Papp, B. (2009) The bryophyte flora of the Aggtelek National Park, In Papp, B. (ed.) The Flora of the Aggtelek National Park. Cryptogams, Natural history of the national parks of Hungary, Magyar Természettudományi Múzeum, Budapest, Vol. 14, 175-230.

85 Papp, B. & Erzberger P. (2010) Contributions to the bryophyte flora of Durmitor National Park, Montenegro, *Nova Hedwigia*, 138, 147-163.

86 Papp, B. & Rajczy, M. (1999) Bryophytes of the Kiskunság National Park and of the other parts of the Danube-Tisza Interfluve. In Lõkös, L., Rajczy, M.(eds.): The flora of the Kiskunság National Park, Cryptogams, Magyar Természettudományi Múzeum, Budapest, 363-413., With correction for Schistidium on the base of Erzberger, P. and Schröder, W. (2008): The genus Schistidium (Grimmiaceae, Musci) in Hungary, Studia Botanica Hungarica, 39, 27-88. Additions from HNHM herbarium collected by Pócs and Boros.

87 Papp, B. & Sabovljević, M. (2002) The bryophyte flora of Tara National Park (W. serbia, Yugoslavia), *Studia botanica Hungarica*, 33, 25-39.

88 Papp, B. & Sabovljević, M. Contribution to the knowledge of the bryoflora of the region of Petnica (West-Serbia) Yugoslavia, *Studia Botanica Hungarica*, 32, 2001, 107-120.

89 Papp, B., Herzberger, P. & Sabovljević, M (2006), contribution to the bryophyte flora of the djerdap national park (E Serbia), *Studia botanica Hungarica*, 37, 131-144.

90 Papp, B., Herzberger, P. & Sabovljević, M. (2004) Contribution to the bryoflora of Kopaonik Mounts (Serbia, Serbie-Montenegro), *Studia Botanica Hungarica*, 35, 67-79.

91 Pistarino, A., Miserere, L., Schumacker, R., D'Andrea, S., Soldan, Z. (2005) Briofite del Piemonte: la collezione della Val Sangone ( Alpi occidentali, Torino), Cataloghi 15, Regione Piemonte museo regionale di scienze Naturali, 456pp.

92 Pokorny, L., Lara, F. & Mazimpaka, V. (2006) The bryophyte Flora of the city of Trento, *Cryptogamie Bryologie*, 27, 2, 265-284.

93 Preston, C.D. (1981) A checklist of Greek liverworts, *Journal of Bryology*, 11, 537-553.

94 Preston, C.D. (1984) A checklist of Greek liverwort: Addendum, *Journal of Bryology*, 13, 97-100.

95 Preston, C.D. (1984) A checklist of Greek mosses, *Journal of bryology*, 13, 43-95.

96 Preston, C.D. & Akeroyde, J.R. (1979) A bryophyte collection from Mt Olympus, Thessaly, Greece, *Revue Bryologique et Lichenologique*, 45, 4, 437-441.

97 Privitera, M. & Puglisi, M. (2002) Some interesting records from the Italian moss flora, *Cryptogamie, Bryologie*, 23, 2, 171-179.

98 Purger, Z., Balogh, L., Papp, B., Rajczy, M. & Szmorad, F. (1997) A Kőszegi-hegység mohaflórája (Bryophyte flora of the Kőszegi Mts), Tilia, 5, 94-272. [with addition for Pohlia from Erzberger, P. (2005), The bulbilliferous species of Pohlia (Bryaceae, Musci) *Studia Botanica Hungarica*, 36, 67-75. - for Schistidium from Erzberger, P. & Schröder, W. (2008), The genus Schistidium (Grimmiaceae, Musci), Studia Botanica Hungarica, 39, 27-88. - for Grimmia on the base Erzberger, P. (2009), The genus Grimmia and Coscinodon (Grimmiaceae, Musci), *Studia Botanica Hungarica*, 40, 37-124.]

99 Rogeon, M.A (1999) Catalogue-Atlas des bryophytes de la Charente, Bulletin de la Société Botanique du Centre-Ouest, Nouvelle série numéro spécial 18, Le clos de la Lande, 61, route de la Lande 17200 Saint-Sulpice-de Royan, ISSN 0759-934X, Bibliothèque de la station scientifique des hautes Fagnes.

100 Sabovljević, M. (2006) Contribution to knowledge of the bryophyte flora of the western alps (Italy, France), *Archives of Biological Sciences*, Belgrade, 58, 1, 61-64.

101 Sabovljević, M. (2006) Contribution to the bryophyte flora of the Djerdap National Park, East Serbia, *Phytologia Balcanica*, 12, 1, 51-54.

102 Sabovljević, M. & Cvetić, T. (2003) Bryoflora of Avala Mounts, central Serbia, Yugoslavia, *Lindbergia*, 28, 90-96.

103 Sabovljević, M. & Grdović, S. (2009) Bryophyte diversity within urban areas: case study of the city of Belgrade (Serbia), *International Journal of Botany*, 5, 1, 85-92.

104 Sabovljević, M. & Sergio, C. (1996) Contribution to the bryflora of Serbia: a bryophyte collection, *Portugaliae Acta Biologica*, 20, 66-74.

105 Sabovljević, M. & Stevanović, V. (1999) Moss conspectus of the Federal republic of Yougoslavia, *Flora Mediterranea*, 9, 65-95.

106 Sabovljević, M., Stevanović, V. (2006) Contribution to the knowledge of the bryophyte flora of Bačka (Vojvodina, Serbia), *Archives of Biological Science Belgrade*, 58, 2, 135-138.

107 Sabovljević, M., Cvetić, T. (2005) A contribution to the bryophyte flora of Fruška Gora, (Vojvodina, Serbia), *Phytologia Balcanica*, 11, 1, 35–43.

108 Sabovljević, M., Tsakiri, E. & Sabovljevića A (2008) Towards the bryophyte flora of Greece, studies in Chalkidiki area (North Greece), *Cryptogamie, Bryologie*, 29, 2, 143-155.

109 Saez, L., Casas, C., Cros, R.M. & Brugues, M. (2002) New bryological data from the balearic islands, *Cryptogamie, Bryologie*, 23, 2, 181-187.

110 Schumacker, R. & Soldán, Z. (2006) Les bryophytes de la vallée d'Aspe ( Parc National des Pyrénnées Atlantiques France), *Lejeunia*, 180, 1-15.

111 Schumacker, R., Soldán, Z., Aleffi, M. & Miserere, L. (1999) The bryophyte flora of the Gran Paradiso National Park (Aosta valley and Piedmont, Italy) and its immediate surroundings: a synthesis, *, Lejeunia*, 160, 1-107.

112 Sergio, C., Brugues, M., Cros, R.M., Garcia, C. & Luro, T. (2006) A new important medditerranean area for bryophyte in Portugal: barrancos(baixo Alantejo), *Boletín de la Sociedad Española de Briología*, 29, 25-33.

113 Shumacker, R. & Sapaly, J. (1997) Catalogue critique des hépatiques ( Anthocerophyta et Marchantiophyta) de l'Auvergne (Cantal et Puy-de-Dôme, France), Documents de la station scientifique des Hautes Fagnes, n°25, Mont Rigi Belgique.

114 SJörs, H. & Een, G. (2000) Wetland bryophytes in Muddus National Park, North Sweden, *Journal of bryology*, 22, 3, 223-236.

115 Sotiaux, A., Pioli, A., Royaud, A., Schumacker, R. & Vanderpoorten, A. (2007) A checklist of the bryophytes of Corsica (France): new records and review of the literature. *Journal of bryology*, 29, 41-53.

116 Sotiaux, A., Stieperaere, H. & Vanderpoorten, A. (2007) Bryophyte checklist and European Red List of the Brussels-Capital region, Flanders and Wallonia (Belgium), *Belgian Jounal of Botany*, vol. 140, 174-196.

117 Stebel, A. (2004) A contribution to the bryoflora of the western part of the Carpathian foothills, From Stebel, A. & Ochyra, R. (eds), *Bryological studies in the Western Carpathians*, Sorus, Poznan, 135-145.

118 Stebel, A. (2004) A contribution to the moss flora of the Gorce (Western Carpathians), From Stebel, A. & Ochyra, R. (eds), *Bryological studies in the Western Carpathians*, Sorus, Poznan, 127-134.

119 Stebel, A. & Bielec, D. (2004) Preliminary studies on the epiphytic bryophytes of Bielsko-Biala town (Western Carpathians). From Stebel, A. & Ochyra, R. (eds), *Bryological studies in the Western Carpathians*, Sorus, Poznan, 147-152.

120 Stefãnut, S., (2008) The Hornwort and liverwort atlas of Romania, Ars Docendi. Univ. Bucuresti press, Bucarest, ISBN 978-973-558-387-3.

121 Szövényi, P., Galambos, I., Hock, Zs. (2001) A Soproni-hegység mohaflórája. (Bryophyte flora of the Soproni Mts), *Tilia*, 10, 5-180.

122 Szurdoki, E., Ódor, P., Papp, B. (2008) A keleméri Mohos-tavak mohaflórája. (Bryophyte flora of Mohos lakes), In Boldogh, S. & G. Farkas, T. (szerk.), A keleméri Mohos-tavak, Kutatás, kezelés, Védelem. (The Mohos peat bogs in Kelemér, Research, Conservation, Management), ANP füzetek IV., Aggteleki Nemzeti Park Igazgatóság, Jósvaf, 65-95. [with addition for Schistidium on the base of Erzberger, P. and Schröder, W. (2008) The genus Schistidium (Grimmiaceae, Musci), *Studia Botanica Hungarica*, 39, 27-88 - for Grimmia on the base Erzberger, P. (2009) The genus Grimmia and Coscinodon (Grimmiaceae, Musci), *Studia Botanica Hungarica*, 40, 37-124.]

123 Thouvenot, L. (2002) Flore bibliographique des bryophytes du département des Pyrénées orientales, France, Catalogne, *Naturalia Ruscinonensia*, 11, 3-72.

124 Townsend, C.C (1977) Bryophytes from some Greek islands, *Revue Bryologique et Lichenologique*, 43, 4, 389-396.

125 Vadam, J-C. (1994) Nouvelles stations Franc-Comtoise de Grimmia teretinervis Limpr., une espèce méconnue en France., *Cryptogamie, Bryologie, Lichenologie*, 15, 2, 153-159.

126 Veljic, M. & Marin, P.D. (1997) New moss taxa for the flora of Serbia, *Flora mediterranea*, 7, 133-138.

127 Villaverde, C., Medina, N.G., Estebanez, B., Medina, R., Mazimpaca, V., infante, M. & Lara, F. (2008) Contribucion al conocimiento de la brioflora del estremo suroeste de la communidad de Madrid, *Boletin de la Societad Espanola de Briologia*, 32-33, 21-29.

128 Vives i Codina, J. (1995) Aproximació a la vegetació briofítica del Baix Solsonés (NE Espanya), *Orsis*, 10, 63-72.

129 Werner, J. (1990) La flore bryologique des environs de Sierck-les-bains(Lorraine) et son intérêt phytogéographique Jean werner, *Cryptogamie, Bryologie, Lichenologie*, 11, 3, 255-266.

130 Werner, J. (1996) Die Moosflora des Luxemburger Oeslings, ministère de la culture travaux scientifiques du Musée National d’Histoire Naturelle du Luxembourg, vol.26, 88pp., ISSN 0251-2424.

131 Werner, J. (1997) Some remarkable bryophytes from Serra da Freita and Serra do Arestal (North Portugal), *Cryptogamie, Bryologie, Lichenologie*, 18, 4, 295-302.

132 Werner, J. (2006) La flore bryologique de la région d'Etretat (Pays de Caux, Haute-Normandie), *Bulletin de la société Géologique de Normandie et des Amis du Museum du Havre*, vol. 93, n°1, 15-37.

133 Zarnowiek, J. & Klama, H. (2004) The bryophyte flora of the Madohora Nature reserve in the Beskid Mali range (Western Carpathians), From Stebel, A. & Ochyra, R. (eds), *Bryological studies in the Western Carpathians*, Sorus, Poznan, 83-100.

134 Zarnowiek, J. & Szwed, W. (2004) A contribution to the moss flora of the subalpine and alpine belts of the Babia Gora massif (Western Carpathians), From Stebel, A. & Ochyra, R. (eds), *Bryological studies in the Western Carpathians*, Sorus, Poznan, 101-118.

20, 40, 53 25SFD

20, 40, 53 26SLH

20, 40, 53 26SMH

20, 40, 53 26SPF

20, 40, 53 26SPG

20, 40, 53 27RYL

20, 40, 53 28RBR

20, 40, 53 28RBS

20, 40, 53 28RCS

20, 40, 53 28RDR

20, 40, 53 28RDS

20, 40, 53 28RES

20, 40, 53 28RFS

20, 40, 53 28RFT

20, 40, 53 28SBB

20, 40, 53 28SCB

21, 24, 34 29SMC

21, 24, 34 29SMD

21, 24, 34 29SNA

21, 24, 34 29SNB

21, 24, 34 29SNC

21, 24, 34 29SND

21, 24, 34 29SNE

21, 24, 34, 112 29SPB

21, 24, 34, 112 29SPC

21, 24, 34 29SPD

21, 24, 34 29SPE

21, 24, 34 29SQA

21, 24, 34 29SQB

21, 24, 34 29SQC

21, 24, 34 29SQD

21, 24, 34 29SQE

21, 24, 34 29TMH

21, 24, 34 29TNE

21, 24, 34, 131 29TNF

21, 24, 34 29TNG

21, 24, 34 29TNH

21, 24, 34 29TNJ

21, 24, 34 29TPE

21, 24, 34 29TPF

21, 24, 34 29TPG

21, 24, 34 29TPH

21, 24, 34 29TPJ

21, 24, 34 29TQE

21, 24, 34 29TQF

21, 24, 34 29TQG

21, 24, 34 29TQH

21, 24, 34 29TQJ

60 29UMT

60 29UMU

60 29UMV

60 29UNA

60 29UNB

60 29UNT

60 29UNU

60 29UNV

60 29UPA

60 29UPB

60 29UPT

60 29UPU

60 29UPV

60 29VND

60 29VPC

60 29VPD

60 29VPE

21, 24, 34 30STE

21, 24, 34 30STF

21, 24, 34 30STG

21, 24, 34 30STH

21, 24, 34 30STJ

21, 24, 34 30SUF

21, 24, 34 30SUG

21, 24, 34 30SUH

21, 24, 34 30SUJ

21, 24, 34 30SUK

21, 24, 34 30SVF

21, 24, 34 30SVG

21, 24, 34 30SVH

21, 24, 34 30SVJ

21, 24, 34 30SVK

34 30SWE

21, 24, 34, 77 30SWF

7, 21, 24, 34 30SWG

7, 21, 24, 34 30SWH

21, 24, 34 30SWJ

21, 24, 34 30SWK

7, 21, 24, 34 30SXG

7, 21, 24, 34 30SXH

21, 24, 34, 52 30SXJ

21, 24, 34, 52 30SXK

21, 24, 34 30SYG

21, 24, 34, 52, 79 30SYH

21, 24, 34, 52 30SYJ

21, 24, 34, 70 30TTK

21, 24, 34 30TTL

21, 24, 34 30TTN

21, 24, 34 30TTP

21, 24, 34, 68, 70, 127 30TUK

21, 24, 34 30TUL

21, 24, 34 30TUM

21, 24, 34, 46, 47, 83 30TUN

21, 24, 34 30TUP

21, 24, 34 30TVK

21, 24, 34, 69, 72 30TVL

21, 24, 34, 51 30TVM

21, 24, 34, 62 30TVN

21, 24, 34 30TVP

15 30TVT

21, 24, 34 30TWK

21, 24, 34 30TWL

21, 24, 34, 23, 75, 76 30TWM

21, 24, 34, 62 30TWN

21, 24, 34, 62 30TWP

21, 24, 34 30TXK

21, 24, 34 30TXL

21, 24, 34, 64, 65 30TXM

21, 24, 34, 43, 45, 50, 62, 110 30TXN

21, 24, 34 30TYK

21, 24, 34, 52 30TYL

21, 24, 34 30TYM

21, 24, 34, 110 30TYN

99 30TYR

60 30UUA

60 30UUB

60 30UUC

60 30UUF

60 30UUG

60 30UVA

60 30UVB

60 30UVC

60 30UVD

60 30UVF

60 30UVG

60 30UWB

60 30UWC

60 30UWD

60 30UWE

60 30UWF

60 30UWG

60 30UXB

60 30UXC

60 30UXD

60 30UXE

60 30UXF

60 30VUH

60 30VUJ

60 30VUK

60 30VVH

60 30VVJ

60 30VVK

60 30VWH

60 30VWJ

21, 24, 34 31SBC

21, 24, 34 31SCC

21, 24, 34, 109 31SCD

21, 24, 34, 109 31SDD

21, 24, 34 31SDE

21, 24, 34, 109 31SED

21, 24, 34 31SEE

21, 24, 34 31SFE

12, 21, 24, 34, 52 31TBE

12, 21, 24, 34, 52 31TBF

21, 24, 34 31TBG

21, 24, 34 31TBH

12, 21, 24, 34, 73 31TCF

12, 21, 24, 34, 64, 65, 128 31TCG

21, 24, 25, 34, 12, 63 31TCH

25 31TCJ

99 31TCL

99 31TCM

12, 21, 24, 34, 73 31TDF

12, 21, 24, 34, 73 31TDG

12, 21, 24, 123 31TDH

25 31TDJ

113 31TDK

113 31TDL

113 31TDM

21, 24, 34 31TEE

12, 21, 24, 34 31TEG

14, 61, 131 31TEK

113 31TEL

38, 114 31TEM

56, 58 31TFJ

39 31TFN

132 31UCR

60 31UCS

60 31UCT

60 31UCU

13 31UDP

55 31UDQ

55 31UDR

55 31UEQ

55 31UER

54, 116, 130 31UFR

28 32SNJ

5, 91 32TLP

5, 57, 91 32TLQ

3, 32, 100, 22, 111 32TLR

81 32TLS

44, 81, 126 32TLT

115 32TMM

115 32TMN

81 32TMR

81 32TMS

81, 78 32TMT

115, 27, 29 32TNL

115 32TNM

115 32TNN

81, GBIF Austria 32TNS

81, 78, GBIFAustria 32TNT

19 32TPN

92 32TPS

78, GBIF Austria 32TPT

54, 78 32ULA

78 32ULB

78 32ULC

78 32ULD

78 32ULE

44 32ULU

78, 129 32ULV

78 32UMA

78 32UMB

78 32UMC

78 32UMD

78 32UME

78 32UMF

78 32UMU

78 32UMV

78 32UNA

78 32UNB

78 32UNC

78 32UND

78 32UNE

78 32UNF

78 32UNU

78 32UNV

78 32UPA

78 32UPB

78 32UPC

78 32UPD

78 32UPE

78 32UPF

78 32UPU

78 32UPV

GBIF scandi 32VKL

GBIF scandi 32VKM

GBIF scandi 32VKN

GBIF scandi 32VKP

GBIF scandi 32VLK

GBIF scandi 32VLL

GBIF scandi 32VLM

GBIF scandi 32VLN

GBIF scandi 32VLP

GBIF scandi 32VLQ

GBIF scandi 32VMK

GBIF scandi 32VML

GBIF scandi 32VMM

GBIF scandi 32VMN

GBIF scandi 32VMP

GBIF scandi 32VMQ

GBIF scandi 32VMR

GBIF scandi 32VNK

GBIF scandi 32VNL

GBIF scandi 32VNM

GBIF scandi 32VNN

GBIF scandi 32VNP

GBIF scandi 32VNQ

GBIF scandi 32VNR

GBIF scandi 32VPJ

GBIF scandi 32VPK

GBIF scandi 32VPL

GBIF scandi 32VPM

GBIF scandi 32VPN

GBIF scandi 32VPP

GBIF scandi 32VPQ

GBIF scandi 32VPR

GBIF scandi 32WNR

GBIF scandi 32WNS

GBIF scandi 32WNT

GBIF scandi 32WPS

GBIF scandi 32WPT

71 33SVB

97 33SWB

6 33SWC

6 33SWD

6 33SXC

6 33SXD

3, 31, 32 33TUH

2 33TUJ

3, 32 33TUM

1, 78, GBIF Austria 33TUN

4 33TVF

3, 32 33TVG

GBIF Austria 33TVM

GBIF Austria 33TVN

GBIF Austria 33TVP

26, 29 33TWE

GBIF Austria 33TWM

GBIF Austria 33TWN

37 33TXM

36, 98, 121 33TXN

GBIF Austria 33TXP

78 33UUA

GBIF scandi 33UUB

GBIF scandi 33UUC

GBIF Austria 33UUP

78 33UUQ

78 33UUR

78 33UUS

78 33UUT

78 33UUU

78 33UUV

78 33UVA

GBIF scandi 33UVB

GBIF scandi 33UVC

GBIF Austria 33UVP

78 33UVS

78 33UVT

78 33UVU

78 33UVV

GBIF Austria 33UWP

GBIF Austria 33UWQ

66 33UXP

GBIF scandi 33VUC

GBIF scandi 33VUD

GBIF scandi 33VUE

GBIF scandi 33VUF

GBIF scandi 33VUG

GBIF scandi 33VUH

GBIF scandi 33VUJ

GBIF scandi 33VUK

GBIF scandi 33VUL

GBIF scandi 33VVC

GBIF scandi 33VVD

GBIF scandi 33VVE

GBIF scandi 33VVF

GBIF scandi 33VVG

GBIF scandi 33VVH

GBIF scandi 33VVJ

GBIF scandi 33VVK

GBIF scandi 33VVL

GBIF scandi 33VWC

GBIF scandi 33VWD

GBIF scandi 33VWE

GBIF scandi 33VWF

GBIF scandi 33VWG

GBIF scandi 33VWH

GBIF scandi 33VWJ

GBIF scandi 33VWK

GBIF scandi 33VWL

GBIF scandi 33VXC

GBIF scandi 33VXD

GBIF scandi 33VXE

GBIF scandi 33VXF

GBIF scandi 33VXG

GBIF scandi 33VXH

GBIF scandi 33VXJ

GBIF scandi 33VXK

GBIF scandi 33VXL

GBIF scandi 33WUM

GBIF scandi 33WUN

GBIF scandi 33WUP

GBIF scandi 33WVL

GBIF scandi 33WVM

GBIF scandi 33WVN

GBIF scandi 33WVP

GBIF scandi 33WVQ

GBIF scandi 33WVR

GBIF scandi 33WVS

GBIF scandi 33WWL

GBIF scandi 33WWM

GBIF scandi 33WWN

GBIF scandi 33WWP

GBIF scandi 33WWQ

GBIF scandi 33WWR

GBIF scandi 33WWS

GBIF scandi 33WWT

GBIF scandi 33WXM

GBIF scandi 33WXN

GBIF scandi 33WXP

GBIF scandi 33WXQ

GBIF scandi 33WXR

GBIF scandi 33WXS

GBIF scandi 33WXT

18 34SFH

18, 95 34SFJ

17 34TCK

17, 30 34TCL

17,30, 33,42 34TCM

126, 67, 105, 85, 33, 104, 42 34TCN

126, 87 34TCP

126, 107 34TCQ

126, 107 34TCR

21, 24, 86, 126 34TCS

17, 21, 24, 17 34TDK

17, 30, 74 34TDL

17, 30, 74, 126 34TDM

90, 126 34TDN

104, 126 34TDP

88, 102, 103, 104, 107, 126 34TDQ

104, 107, 106, 120, 126 34TDR

35, 84, 122 34TDU

93, 94, 95 34TEK

74 34TEL

74, 126 34TEM

126 34TEN

126 34TEP

89, 101, 120,126 34TEQ

126, 120 34TER

120 34TES

120 34TET

96, 108 34TFK

48, 49, 74 34TFL

49, 48, 80, 126 34TFN

21, 24, 120, 127 34TFP

89, 120, 127 34TFQ

80, 120 34TFR

80, 120 34TFS

120 34TFT

18, 59, 93, 94, 95 34TGK

117, 119, 133 34UCA

41 34UCF

134 34UCV

117 34UDA

111 34UDB

118 34UDV

8, 9, 10 34UEA

GBIF scandi 34VCJ

GBIF scandi 34VCK

GBIF scandi 34VCL

GBIF scandi 34VCM

GBIF scandi 34VCN

GBIF scandi 34VCQ

GBIF scandi 34VCR

GBIF scandi 34VDM

GBIF scandi 34VDR

GBIF scandi 34VEM

GBIF scandi 34VEN

GBIF scandi 34VEP

GBIF scandi 34VFM

GBIF scandi 34VFN

GBIF scandi 34VFP

GBIF scandi 34VFQ

GBIF scandi 34WCB

GBIF scandi 34WCC

GBIF scandi 34WCS

GBIF scandi 34WCT

GBIF scandi 34WCU

GBIF scandi 34WCV

GBIF scandi 34WDA

GBIF scandi 34WDB

GBIF scandi 34WDC

GBIF scandi 34WDR

GBIF scandi 34WDS

GBIF scandi 34WDT

GBIF scandi 34WDU

114, GBIF scandi 34WDV

GBIF scandi 34WEA

GBIF scandi 34WEB

GBIF scandi 34WEC

GBIF scandi 34WED

GBIF scandi 34WES

GBIF scandi 34WET

GBIF scandi 34WEU

GBIF scandi 34WEV

GBIF scandi 34WFA

GBIF scandi 34WFB

GBIF scandi 34WFC

GBIF scandi 34WFD

GBIF scandi 34WFT

GBIF scandi 34WFU

GBIF scandi 34WFV

124 35SLA

124 35SLB

16 35SLU

124 35SMB

16 35SMU

124 35SNA

124 35TLF

49 35TLG

49 35TLH

80 35TLJ

120 35TLK

80, 120 35TLL

120 35TLM

80, 120 35TLN

120 35TMJ

120 35TMK

120 35TML

120 35TMM

120 35TMN

120 35TNJ

120 35TNK

120 35TNL

120 35TNM

120 35TNN

120 35TPJ

120 35TPK

120 35TPL

GBIF scandi 35VLG

GBIF scandi 35VLH

GBIF scandi 35VLJ

GBIF scandi 35VLK

GBIF scandi 35VMG

GBIF scandi 35VMH

GBIF scandi 35VMJ

GBIF scandi 35VMK

GBIF scandi 35VNF

GBIF scandi 35VNH

GBIF scandi 35VNJ

GBIF scandi 35VNK

GBIF scandi 35VNL

GBIF scandi 35VPJ

GBIF scandi 35VPK

GBIF scandi 35VPL

GBIF scandi 35WLM

GBIF scandi 35WLP

GBIF scandi 35WLT

GBIF scandi 35WLU

GBIF scandi 35WML

GBIF scandi 35WMM

GBIF scandi 35WMN

GBIF scandi 35WMP

GBIF scandi 35WMQ

GBIF scandi 35WMS

GBIF scandi 35WMT

GBIF scandi 35WMU

GBIF scandi 35WNL

GBIF scandi 35WNN

GBIF scandi 35WNQ

GBIF scandi 35WNS

GBIF scandi 35WNT

GBIF scandi 35WNU

GBIF scandi 35WPP

GBIF scandi 35WPS

GBIF scandi 35WPT

82 36UVG

82 36UWG

82 36VUH

GBIF scandi 36VUQ

82 36VVH

82 36VVJ

82 36VWH

82 36VWJ

82 36VWK

GBIF scandi 36WUC

GBIF scandi 36WUD

GBIF scandi 36WV
