# Supplementary material for: Modeling Species Distributions from Heterogeneous Data for the Biogeographic Regionalization of the European Bryophyte Flora
Source: PLoS One. 2013 Feb 11;8(2):e55648. doi: 10.1371/journal.pone.0055648 (PMC3569459; doi:10.1371/journal.pone.0055648)
Supplement: Appendix S1 — Nomenclature followed for moss and liverwort species with corresponding references. (DOC) [file pone.0055648.s003.doc]

APPENDIX S1. Nomenclature followed for mosses and liverworts species recognition with corresponding references.

Nomenclature after Grolle & Long (2000) for liverworts and Hill et al. (2006) for mosses, except:

- *Hygroamblystegium varium* was considered in a broad sense (incl. *H. fluviatile, H. tenax*, and *H. humile*) following Vanderpoorten (2004) because of the confusion and differences in taxonomic interpretations of the species of this complex;

- *Racomitrium canescens* was considered in a broad sense (incl. *R. ericoides* and *R. elongatum*) because Frisvoll’s (1983) taxonomic revision has not been followed in a uniform way across Europe. The recent acceptation of the segregate species of *R. canescens* in some countries (e.g., Soltés 2008) inevitably lead to differences in the knowledge of their distribution at the European scale. The same was done for the *R. heterostichum* complex, so that the distributions of *R. affine, R. microcarpon* and *R.* *sudeticum* were merged with those of *R.* *heterostichum* s.l.

- *Aloina aloides* and *Plagiothecium nemorale* were considered in a broad sense (incl. *A. ambigua* and *P. succulentum*, respectively) because, although the two species are listed as distinct in Hill’s et al. (2006) checklist, they are elsewhere considered as synonyms (e.g., Dirkse et al. 1999), leading to potential confusions in their actual distributions.

- The distributions of *Schistidium* species were not recorded: Blom’s (1996) revision, supported by molecular evidence (Goryunov et al. 2007), suggests that species, as traditionally defined, were polyphyletic. Blom’s taxonomic system has, however, not been applied everywhere followed across Europe, making it eventually imposible to homogenize taxonomic concepts in the group across countries and obtain reliable distributions for the different taxa.

- *Chiloscyphus* *polyanthos* was considered in a broad sense (incl. *C. pallescens*) because, although *C. pallescens* and *C. polyanthos* exhibit clearly different characters in the reproductive system (Paton 1999), they are mostly found sterile, challenging their identification.

- *Polytrichum commune* was considered in a broad sense (incl. *P. uliginosum*) because, although *P. uliginosum* and *P. commune* exhibit clear genetic and morphological differences that support species recognition (Bijlsma et al. 2000), they are still considered as synonyms in many areas, precluding to achieve a good knowledge of their distributions at the European scale.

**References**

Bijlsma, R., M. van der Velde, L. van de Zande, A. C. Boerema, B. O. van Zanten, 2000: Molecular Markers Reveal Cryptic Species Within Polytrichum commune (Common Hair-Cap Moss). Plant Biology, Volume 2, Issue 4, pages 408–414.

Blom H.H. 1996. A Revision of the Schistidium apocarpum complex in Norway and Sweden. Bryophytorum Bibliotheca 49 : 1-334.

Dirkse G., During H. & Siebel H. 1999. Standaardlijst van de Nederlandse blad-, lever- en hauwmossen. Buxbaumiella 50 : 68-128.

Frisvoll A. (1983): A taxonomic revision of the *Racomitrium canescens* group (Bryophyta, Grimmiales). Gunneria 41: 1-181.

Goryunov, D. V.; Ignatova, E. A.; Ignatov, M. S.; Milyutina, I. A.; Troitsky, A. V. 2007. Support from DNA data for a narrow species concept in Schistidium (Grimmiaceae, Musci). J. Bryol. 29 : 98-103.

Paton J.A. 1999. The Liverwort Flora of the British Isles. Apollo Books.

Soltés R. 2008. *Racomitrium elongatum* Frisvoll, a neglected Slovakian bryophyte. Thaiszia 18 : 59-64.

Vanderpoorten, A. 2004. A simple taxonomic treatment for a complicated evolutionary story: the genus *Hygroamblystegium*. Monographs in Systematic Botany 98: 320--327.
